# Intimate Partner Violence: A form of Trauma-Coerced Attachment?

**DOI:** 10.1192/j.eurpsy.2026.12050

**Published:** 2026-07-31

**Authors:** D. Agius, D. Vella Fondacaro

**Affiliations:** Department of Psychiatry, University of Malta, Msida, Malta

## Abstract

**Introduction:**

Intimate partner violence (IPV) remains a significant public health and legal concern, particularly in Malta, where rates of gender-based violence and femicide are alarming. Victims may withdraw police reports, minimise the threat they face, or defend their abusers—even after seeking help. These behaviours are often misunderstood as ambivalence or consent. Emerging literature suggests they may reflect a complex psychological phenomenon known as trauma-coerced attachment: a pathological bond formed under conditions of fear, coercion, and control. Over time, psychological boundaries may erode, contributing to identity disturbance consistent with the dissociative category of “prolonged and intense coercive persuasion” described in DSM-5 under OSDD.

**Objectives:**

This study explores how pathological emotional bonds may develop between victims and perpetrators of IPV, and how such connections influence self-perception, cognitive dissonance, and emotionally conflicted decision-making. It aims to understand survivors’ psychological experiences in depth—regardless of alignment with existing trauma models—toward developing more trauma-informed approaches in clinical and legal contexts.

**Methods:**

This qualitative study involves semi-structured interviews with adult female participants (18+) who experienced IPV and exited the relationship at least six months prior. Interviews are conducted in Maltese or English, lasting 60–90 minutes. Participants are recruited via Victim Support Agency (VSA), a local NGO. All interviews are audio-recorded with consent, anonymised, and analysed thematically. While interviews may be transcribed in either language, the full analysis and write-up are conducted in English. Data is processed in accordance with GDPR, and psychological support is offered throughout.

**Results:**

The study is currently in the data collection phase. Thematic analysis will explore how participants describe identity shifts, emotional dependency, ambivalence, and interpretations of their experience post-abuse. Particular focus is given to decisions to stay or leave, internalised coercion, and evolving self-concept. Although frameworks like trauma-coerced attachment and OSDD inform the study, analysis remains exploratory. Regardless of alignment, findings are expected to enhance our understanding of survivors’ psychological responses to IPV.

**Conclusions:**

Findings are expected to deepen insight into how survivors interpret and reconstruct their experience following coercive relationships. This knowledge may support more accurate and compassionate approaches in psychiatry, victim advocacy, and legal interpretation—particularly in cases where behaviour is misjudged as inconsistent or contradictory.

**Disclosure of Interest:**

None Declared

